# Associations between meniscal tears and various degrees of osteoarthritis among dogs undergoing TPLO for cranial cruciate ligament rupture

**DOI:** 10.1186/s13104-023-06307-0

**Published:** 2023-03-13

**Authors:** Canny Fung, Michael Ficklin, Chika C. Okafor

**Affiliations:** 1 Blue Pearl Veterinary Partners, 1646 Spring Cypress Rd, 77388 Spring, TX United States of America; 2 Department of Biomedical and Diagnostic Sciences, College of Veterinary Medicine, University of Tennessee at Knoxville, Room A 326 Veterinary Medical Center Building, 2407 River Drive, 37996 Knoxville, TN United States of America

**Keywords:** Stifle, meniscus, Osteoarthritis, Canine

## Abstract

**Objective:**

The aim of this study was to evaluate the association between meniscal lesions and severity of osteoarthritis (OA) among dogs that underwent Tibial Plateau Leveling Osteotomy (TPLO) for stabilization of cranial cruciate ligament rupture (CrCLR) at the University of Tennessee in 2011–2017.

**Results:**

There were a total of 252 meniscal tears. Factors associated with diagnosis of medial meniscal tears (MMT) in dogs were severe OA in comparison to no OA (3.8 OR, 2.0–8.0 95% CI, 0.001 p-value), sporting and mixed breed group compared to other breed (3.6 OR, 1.7–7.6 95% CI, 0.004 p-value; 3.2 OR, 1.6–6.6 95% CI, 0.019 p-value, respectively), increasing age (1.1 OR, 1.0-1.2 95% CI, 0.018 p-value), complete CrCLR compared to partial (3.3 OR, 2.1-5.0 95% CI, < 0.001 p-value), and arthrotomy compared to arthroscopy (2.2 OR, 1.4–3.1 95% CI, 0.002 p-value). The factors that did not have significance in predicting MMT were weight, sex, lameness period, and side affected.

## Introduction

Osteoarthritis (OA) is a progressive disease characterized by joint effusion, osteophytes and enthesophytes, intra-articular mineralization, and less reliably subchondral sclerosis. [1–3] In the stifle, a proposed mechanism of the development of OA from meniscal injury is through the release of pro-catabolic and pro-algesic enzymes, cytokines, chemokines, DAMPs and matricyptins. [1, 4–8] Therefore, accurate identification of meniscal pathology is essential as undiagnosed meniscal pathology may result in persistent lameness, pain, and dysfunction and is associated with an increased risk of developing OA. [4,9] Various methods of diagnosing meniscal pathology have been evaluated, including advanced imaging modalities and surgery. [5,10,11]

While Kaufman et al. [15] evaluated the prevalence of meniscal tears using arthroscopy and found no significant relationship between periarticular osteophytosis and meniscal injury, no study has specifically evaluated the association of varying degrees of OA and meniscal tears in dogs. The purpose of this retrospective study is to evaluate the associations of meniscal pathology in dogs with varying degrees of degenerative joint disease undergoing arthrotomy or arthroscopy prior to TPLO stabilization. We hypothesize that the prevalence of meniscal tears is high with severe OA.

## METHODS

### Study design and setting

A retrospective, cross-sectional study was performed using information retrieved from the medical records of dogs undergoing surgical repair for CrCLR with TPLO. Medical records of these dogs from the University of Tennessee, between 2011 and 2017, were reviewed. Data retrieved from the medical records include signalment consisting of age in years, sex (female intact or spayed or male intact or castrated), breed group (herding, mixed, sporting, working or other (terrier, toy, non-sporting and hound)), weight in kilogram, lameness period (onset of lameness to surgery, acute < 30 days, subacute 31–150 days, chronic > 150 days), the hind limb affected (left or right), evaluation of the joint via arthroscopy or arthrotomy (medial parapatellar), level of OA (none, mild, moderate or severe) based on a subjective grading score validated by the method described by Brunnberg et al. (1992) [33], meniscal pathology (intact or torn and type of tear; bucket handle, unspecified, complex, radial, degenerative, vertical longitudinal, flap) at the time of surgery and treatment (removed or released and type of meniscectomy including partial, total or hemi), concurrent surgical procedures, and complications intra-operatively or during follow-up. A medical record had to report entry in all the aforementioned variables of interest to be included in the study. Data were maintained in standard spreadsheet format (Microsoft Excel; Microsoft USA).

### Statistical analysis

Statistical analyses comparing medial meniscal injuries, presence, and severity of OAs duration of clinical signs, signalment, arthroscopy, or arthrotomy were performed using SAS 9.4 software. Descriptive statistics (frequencies, proportions, median, mean ± SD, range) were used to summarize the measured explanatory variables. Univariable logistic regression was used to test associations between each explanatory variable and the outcome of interest. Odds ratios and their 95% CIs were used to measure the strength of associations between the explanatory variables and the outcome. A *P*value of ≤ 0.05 was considered significant. In preparation for building a multivariable model, the assumption of linearity of age and body weight was tested by examining the statistical significance of using a squared term. If there was a quadratic relationship, the independent variable’s squared term was considered for addition to the model.

All the variables in the univariable analyses were explored in the model building and interactions between selected variables were tested. The tested interactions include age and OA; age and breed group; age and sex. A non-intervening variable that changed the coefficient of a previously significant variable in the logarithm scale by at least 20% was treated as a confounder and was retained in the model. The final model included explanatory variables that remained significant.

## RESULTS

The records search identified 479 stifles that underwent TPLO between 2011 and 2017 (Table 1). Of these 479 stifles, 80 were bilateral procedures. Dog ages ranged from 0.8 years to 15 years (median 5) and weight ranged from 3.10 to 80.50 kg (median 33); the distribution of dog ages and weights by their meniscus tear status is presented in Table 2. In total, there were 66 breeds categorized into their respective breed groups. The most common breeds were mixed 161 (34%), sporting 134 (28%), working 87 (18%), and herding 43 (9%). The breed groups terrier, non-sporting, toy, and hound were combined and labeled as other 53 (11%). The sex and onset of lameness frequency data are presented in Table 1.

**Table 1:** Summary data of the distribution of all continuous variables of dogs presented to the University of Tennessee from 2011–2017 for cranial cruciate ligament disease stabilization.

Variable	N	Min	Lower quartile	Mean	95% CL for mean	Std Dev	Median	Upper Quartile	Max
Lateral meniscal tear = Torn									
Age (yr)	8	3.0	4.5	6.0	4.2–7.8	2.1	6.0	7.5	9.0
Weight (Kg)	5	19.0	22.5	39.8	11.9–67.7	22.5	31.3	55.0	71.2
Lateral meniscal tear = Intact									
Age (yr)	471	0.8	3.0	5.6	5.3–5.8	2.7	5.0	7.0	15.0
Weight (Kg)	410	3.1	26.8	34.3	33.1–35.5	12.2	33.0	40.0	80.5
Medial meniscal tear = Torn									
Age (yr)	244	1.0	4.0	6.1	5.7–6.4	2.7	6.0	8.0	15.0
Weight (Kg)	209	3.1	27.4	35.1	33.5–36.7	11.4	33.8	44.0	74.0
Medial meniscal tear = Intact									
Age (yr)	235	0.8	3.0	5.1	4.7–5.4	2.6	5.0	7.0	12.0
Weight (Kg)	206	5.9	25.9	33.6	31.8–35.4	13.2	31.9	40.4	80.5

**Table 2:** Summary data of the frequency of all categorical variables of dogs presenting to the University of Tennessee from 2011–2017 for cranial cruciate ligament disease stabilization.

	Frequency		Medial Meniscal Tear		Lateral Meniscal Tear	
Variable	Number	Percent %	Torn	Intact	Torn	Intact
Sex						
Female						
Spayed	232	48	121	111	4	228
Intact	9	2	5	4	0	9
Male						
Castrated	216	45	105	111	4	212
Intact	22	5	13	9	0	22
Breed Group						
Mixed	161	34	94	67	1	160
Sporting	134	28	80	54	1	133
Working	88	18	35	53	2	86
Herding	43	9	19	24	2	41
Other (Terrier, Non-Sporting,	53	11	16	37	2	51
Toy, Hound)						
Lameness Period (days)						
Acute (< 30 days)	262	55	133	129	4	258
Subacute (31–150 days)	115	24	56	59	2	113
Chronic (> 151 days)	102	21	55	47	2	100
Side						
Left	223	47	112	111	3	220
Right	255	53	132	123	5	250
Both	1	1	0	1	0	1
Procedure						
Arthroscopy	150	31	59	91	6	144
Arthrotomy	320	67	181	139	2	318
Both	9	2	4	5	0	9
CrCLR						
Complete	310	65	189	121	5	305
Partial	169	35	55	114	3	166
Osteoarthritis Severity						
None	163	34	73	90	3	160
Mild	173	36	88	85	3	170
Moderate	82	17	40	42	1	81
Severe	61	13	43	18	1	60

Arthrotomy was performed in 320 (67%) stifles, arthroscopy in 150 (31%) stifles, and 9 (2%) had both procedures performed. Both procedures were performed in cases that required conversion from arthroscopy to arthrotomy due to technical difficulties. 65% (310) of stifle joints had complete CrCLR whereas 35% (169 joints) had partial tears. 50% (244) of joints had a medial meniscal tear, and 49% (235) were intact. The most common type of medial meniscal tear was vertical longitudinal (39%, 94), followed by bucket handle 50 (20%), unspecified 44 (18%), complex 20 (8%), radial 14 (6%), degenerative 21 (9%), and flap 1 (0%). The meniscus was removed in 226 (47%) and released in 37 (8%). Of the removed menisci, partial meniscectomy was performed in 116 (51%), hemimeniscectomy was performed in 64 (28%) and a total meniscectomy was performed in 48 (21%). The lateral meniscus was intact in 471 (98%,) and torn in 8 (2%). There was no major difference in the hindlimb affected, right 255 (53%) and left 223 (47%). 36% (173) had mild OA, 17% (82) had moderate, 13% (61) had severe, and 34% (163) had none. Repeat surgery was performed in 34 (7%). Of these surgeries, 8 (24%) were due to a meniscal tear and 26 (76%) were not. Surgical complications were noted in 149 (31%).

Based on the univariable logistic regression model (Table 3), age, breed, surgical procedure, CrCLR, and OA severity were potential predictors of medial meniscal tears in dogs presenting for cranial cruciate ligament disease stabilization. Increasing age, at one-year intervals, (1.15 OR, 1.1–1.2 95% CI, < 0.001 p-value) was associated with MMT. Compared to other dogs, sporting dogs (3.4 OR, 1.7–6.8 95% CI, 0.002 p-value) and mixed breed dogs (3.2 OR, 1.7–6.3 95% CI, 0.003 p-value) were associated with MMT. Compared to arthroscopy, arthrotomy (2.0 OR, 1.4-3.0 95% CI, 0.001 p-value) revealed more meniscal tears. Compared to partial CrCLR, complete CrCLR (3.1 OR, 2.1–4.8 95% CI, < 0.001 p-value) was associated with MMT. Compared to no OA, severe OA (3.0 OR, 1.6–5.5 95% CI, 0.002 p-value) was associated with the presence of meniscal tears. Weight (P = 0.274), sex (P = 0.554), lameness period (P = 0.741), and side (P = 0.737) were not found to be statistically significant and were excluded from the multivariable logistic regression model.

**Table 3:** Results of Univariable and Multivariable logistic regression models used to assess potential predictors of medial meniscal tears of dogs presented to the University of Tennessee from 2011–2017 for cranial cruciate ligament disease stabilization

Univariable	Med Meniscal Tear		
Variable	OR	95% CI	p-value
Age (1 year increase)	1.15	1.1–1.2	< 0.001
Sex			
Female vs Male	1.1	0.8–1.6	0.554
Weight (1 kg increase)	1	0.9–1.1	0.208
Breed Group			0.001
Mixed	3.2	1.7–6.3	0.003
Sporting	3.4	1.7–6.8	0.002
Working	1.5	0.7–3.2	0.187
Herding	1.8	0.8–4.2	0.752
Other (Terrier, Non-Sporting,	Ref	-	-
Toy, Hound)			
Lameness Period (days)			0.741
Acute (< 30 days)	1.1	0.7–1.7	0.906
Subacute (31–150 days)	Ref	-	-
Chronic (> 151 days)	1.2	0.7–2.1	0.462
Side			
Left vs Right	1.1	0.7–1.5	0.737
Procedure			
Arthrotomy	2	1.4-3.0	0.001
Arthroscopy	Ref	-	-
CrCLR			
Complete	3.1	2.1–4.8	< 0.001
Partial	Ref	-	-
Osteoarthritis Severity			0.01
None	Ref	-	-
Mild	1.3	0.8-2.0	0.397
Moderate	1.2	0.7–1.2	0.263
Severe	3	1.6–5.5	0.002
Multivariable	Medial Meniscal Tear		
Variable	OR	95% CI	p-value
Age (1 year increase)	1.1	1.0-1.2	0.018
Breed Group			0.003
Mixed	3.2	1.6–6.6	0.019
Sporting	3.6	1.7–7.6	0.004
Working	1.8	0.8-4.0	0.495
Herding	2	0.8–4.9	0.809
Other (Terrier,	Ref	-	-
Non-Sporting,			
Toy, Hound)			
Procedure			
Arthrotomy	2.2	1.4–3.1	0.002
Arthroscopy	Ref	-	-
CrCLR			
Complete	3.3	2.1-5.0	< 0.001
Partial	Ref	-	-
Osteoarthritis Severity			0.003
None	Ref	-	-
Mild	1.4	0.8–2.2	0.475
Moderate	1.1	0.6–1.9	0.081
Severe	3.8	2.0–8.0	0.001

After the multivariable logistic regression model, (Table 3), age, breed (sporting dogs and mixed breed dogs), surgical procedure (arthrotomy), CrCLR (complete), and OA severity remained significantly associated with the occurrence of medial meniscal tears in dogs presenting for CrCLR stabilization. None of the tested interactions was significant and no confounder was detected.

## DISCUSSION

This study reveals that dogs undergoing TPLO with severe OA had a greater prevalence of MMT. The odds of this association increased with complete CrCLR vs. partial CrCLR. In addition, we demonstrated a higher frequency of MMT in sporting and mixed-breed groups which is also supported by other studies reporting breed differences in the prevalence of MMT. [17, 21] We also found increasing age was positively associated with MMT. Additionally, we found a higher incidence of MMT diagnosed with arthrotomy compared to arthroscopy but this may be due to a higher caseload where arthrotomy (350;67%) was performed compared to arthroscopy (150;31%). The prevalence of these differences persists through the multivariate analysis and also showed that there were no significant differences found in the weight, sex, side of injury, and surgery period which was consistent with previous studies. [16, 30]

The prevalence of MMT was found to be 50% in this study, which is similar to that of previously reported studies. [5,12,16] The most consistent reported statistical finding influencing MMT, and reinforced in this study, was the association with complete CrCLR. [16–21] With altered stifle biomechanics due to MMT and instability associated with CrCLR, it is well recognized that the progression of OA accelerates but the sequence of events is still unclear. [1,3,20,22] Osteoarthritis can be both a consequence and a cause of MMT. [15,22] The association between MMT and higher degrees of OA is evident and may be due to the loss of meniscal function causing increased progression of osteophytes. Proinflammatory mediators in the joint drive degradative processes in the menisci, and the menisci contribute to OA through their own production of proinflammatory and degradative mediators. [6–8] The increases in IL-6, IL-8, MCP-1, and KC (GROα) by the meniscus in response to IL1-β stimulation is similar to that reported for chondrocytes and synoviocytes, indicating that the meniscus is another source for these cytokines and chemokines during OA development and progression. [6]–8] It is also well known that OA occurs secondary to the loss of function of CrCLR and can therefore affect all tissues in the joint to varying degrees, including the meniscus. [14] Stifle OA can also contribute to MMT by abnormal loading and breakdown of meniscal structure. [14,22,24,25]

We evaluated OA in CrCLR with and without MMT via arthroscopy and arthrotomy and found MMT occurred more frequently with severe OA, but not with mild or moderate OA. Our findings are consistent with previous reports that the severity of OA did not correlate with the severity of the meniscal injury but that meniscal injury itself did correlate with severe OA. [17,26] Contrary to Flo 1993, Gambaredella 1981, Timmermann 1998 *and* Kaufman 2017, our study did not show an association of MMT with a duration of lameness. [15, 27−29] Differences can include the definition of duration of lameness; we evaluated the duration of lameness as the length of time between the onset of lameness and surgery and categorized them into groups of subacute, acute, and chronic.

The normal aging process leads to progressive loss of collagen fiber organization, decreased cell function, reduced cell density, and loss of water content. With the inability to effectively maintain the extracellular matrix, the meniscus experiences oxidative stress and damage through modulation of cell signaling pathways that regulate anabolic and catabolic activity resulting in abnormal matrix organization and cellularity. [34,35]. This structural disorganization can then lead to meniscal injury which is consistent with our findings and others that increasing age is a risk factor for MMT [23, 34−37]

Studies have shown that meniscal injuries are a common postoperative complication of surgical stabilization in dogs treated for CrCLR. [13,31,32] The presence of post-surgical meniscal pathology may be due to failure of diagnosis at the time of surgery (latent tears) or a result of residual joint instability (post-liminary tears). [13,32] In this study, we found a 24% chance of repeat surgery due to latent or post-liminary MMT. Therefore, intra-operatively, it would be beneficial to further focus on meniscus pathology when given a stifle with severe OA, increasing age, a sporting, and mixed breed group, and when the CrCLR is complete.

## LIMITATIONS

The limitations of the study are that it is retrospective in nature with interobserver variability in the grading of OA. Other factors that could affect this data include the surgeon’s skill level for detecting meniscal pathology, the surgeon’s skill level and preference for arthroscopy vs. arthrotomy, latent meniscal tears which were missed at initial arthrotomy (and which may have been diagnosed with arthroscopy), and insufficient record keeping.

## Data Availability

The datasets used and/or analyzed during the current study are available from the corresponding author on reasonable request.

## List of abbreviations

TPLO: Tibial plateau leveling osteotomy
OA: Osteoarthritis
OR: Odds ratio
CrCLR: Cranial cruciate ligament rupture
MMT: Medial meniscal tear

## References

[1] Kahn D, Mittelstaedt D, Matyas J, et al. Meniscus Induced cartilaginous damage and non-linear gross anatomical progression of early-stage Osteoarthritis in a Canine Model. Open Orthop J. 2016;248690–705. 10.2174/1874325001610010690 10.2174/1874325001610010690 PMC5220181 28144379

[2] D’Anjou MA, Moreau M, Troncy É (2008). Osteophytosis, subchondral bone sclerosis, joint effusion and soft tissue thickening in canine experimental stifle osteoarthritis: comparison between 1.5 T magnetic resonance imaging and computed radiography. Vet Surg.

[3] Widmer WR, Buckwalter KA, Braunstein EM (1994). Radiographic and magnetic resonance imaging of the stifle joint in experimental osteoarthritis of dogs. Vet Radiol Ultrasound.

[4] Melrose J, Fuller ES, Little CB (2017). The biology of meniscal pathology in osteoarthritis and its contribution to joint disease: beyond simple mechanics. Connect Tissue Res.

[5] Mahn MM, Cook JL, Cook CR (2005). Arthroscopic verification of ultrasonographic diagnosis of meniscal pathology in dogs. Vet Surg.

[6] Goldring MB, Otero M, Plumb DA (2011). Roles of inflammatory and anabolic cytokines in cartilage metabolism: signals and multiple effectors converge upon MMP-13 regulation in osteoarthritis. Eur Cell Mater.

[7] Golds EE, Mason P, Nyirkos P (1989). Inflammatory cytokines induce synthesis and secretion of Gro protein and a Neutrophil Chemotactic factor but not Beta 2-Microglobulin in human synovial cells and fibroblasts. Biochem J.

[8] Hennerbichler A, Moutos FT, Hennerbichler D (2007). Interleukin-1 and tumor necrosis factor alpha inhibit repair of the porcine meniscus in vitro. Osteoarthritis Cartilage.

[9] Ritzo ME, Ritzo BA, Siddens AD (2014). Incidence and type of Meniscal Injury and Associated Long-Term Clinical Outcomes in Dogs treated surgically for cranial cruciate ligament disease. Vet Surg.

[10] Samii VF, Dyce J, Pozzi A (2009). Computed tomographic arthrography of the stifle for detection of cranial and caudal cruciate ligament and meniscal tears in dogs. Vet Radiol Ultrasound.

[11] Martig S, Konar M, Schmökel HG (2006). Low-field mri and arthroscopy of meniscal lesions in ten dogs with experimentally induced cranial cruciate ligament insufficiency. Vet Radiol Ultrasound.

[12] Pozzi A, Hildreth B, Rajala-Schultz P (2008). Comparison of arthroscopy and arthrotomy for diagnosis of Medial Meniscal Pathology: an Ex vivo study. Vet Surg.

[13] Thieman KM, Tomlinson JL, Fox DB (2006). Effect of meniscal release on rate of subsequent meniscal tears and owner-assessed outcome in dogs with cruciate disease treated with tibial plateau leveling osteotomy. Vet Surg.

[14] Lohmander LS, Englund PM, Dahl LL (2007). The long-term consequence of anterior cruciate ligament and meniscus injuries: Osteoarthritis. Am J Sports Med.

[15] Kaufman K, Beale BS, Thames HD (2017). Articular cartilage scores in cranial cruciate ligament-deficient dogs with or without bucket handle tears of the medial meniscus. Vet Surg.

[16] Ralphs SC, Whitney WO (2002). Arthroscopic evaluation of menisci in dogs with cranial cruciate ligament injuries: 100 cases (1999–2000). J Am Vet Med Assoc.

[17] Neâas A, Zatloukal J (2002). Factors related to the risk of Meniscal Injury in Dogs with Cranial Cruciate Ligament rupture. Acta Vet Brno.

[18] Voss K, Damur DM, Guerrero T (2008). Force plate gait analysis to assess limb function after Tibial Tuberosity Advancement in Dogs with Cranial Cruciate Ligament Disease. Vet Comp Orthop Traumatol.

[19] Casale SA, McCarthy RJ (2009). Complications associated with lateral fabellotibial suture surgery for cranial cruciate ligament injury in dogs: 363 cases (1997–2005). J Am Vet Med Assoc.

[20] Shimada M, Mizokami N, Ichinohe T (2020). Long-term outcome and progression of osteoarthritis in uncomplicated cases of cranial cruciate ligament rupture treated by tibial plateau leveling osteotomy in dogs. J Vet Med Sci.

[21] Hayes GM, Langley-Hobbs SJ, Jeffery ND (2010). Risk factors for medial meniscal injury in association with cranial cruciate ligament rupture. J Small Anim Pract.

[22] Englund M, Guermazi A, Lohmander SL (2009). The role of the Meniscus in knee osteoarthritis: a cause or Consequence?. Radiol Clin North Am.

[23] Krupkova O, Smolders L, Wuertz-Kozak K (2018). The pathobiology of the meniscus: a comparison between the human and dog. Front Vet Sci.

[24] Christoforakis J, Pradhan R, Sanchez-Ballester J (2005). Is there an Association between articular cartilage changes and degenerative Meniscus tears?. Arthroscopy.

[25] Lange AK, Fiatarone Singh MA, Smith RM (2007). Degenerative meniscus tears and mobility impairment in women with knee osteoarthritis. Osteoarthritis Cartilage.

[26] Smith GN, Mickler EA, Albrecht ME (2002). Severity of medial meniscus damage in the canine knee after anterior cruciate ligament transection. Osteoarthritis Cartilage.

[27] Flo GL (1993). Meniscal injuries. Vet Clin North Am Small Anim Pract.

[28] Gambardella PC, Wallace LJ, Cassidy F (1981). Lateral suture technique for management of Anterior Cruciate Ligament rupture in dogs: a retrospective study. J Am Anim Hosp Assoc.

[29] Timmermann C, Meyer-Lindenberg A, Nolte I (1998). Meniscus injuries in dogs with rupture of the cruciate ligament. Dtsch Tierarztl Wochenschr.

[30] Mccready DJ, Ness MG (2016). Diagnosis and management of meniscal injury in dogs with cranial cruciate ligament rupture: a systematic literature review. J Small Anim Pract.

[31] Bennett D, May C (1991). Meniscal damage associated with cruciate disease in the dog. J Small Anim Pract.

[32] Case JB, Hulse D, Kerwin SC (2008). Meniscal injury following initial cranial cruciate ligament stabilization surgery in 26 dogs (29 stifles). Vet Comp Orthop and Traumatol.

[33] Brunnberg L, Rieger I, Hesse E (1992). 7 years experience with modified over-the-top crucial ligament plastic operation in dog. Kleintierpraxis.

[34] Loeser RF (2009). Aging and osteoarthritis: the role of chondrocyte senescence and aging changes in the cartilage matrix. Osteoarthritis Cartilage.

[35] Pauli C, Grogan SP, Patil S, Otsuki S, Hasegawa A, Koziol J (2011). Macroscopic and histopathologic analysis of human knee menisci in aging and osteoarthritis. Osteoarthritis Cartilage.

[36] Jackson J, Vasseur PB, Griffey S (2001). Pathologic changes in grossly normal menisci in dogs with rupture of the cranial cruciate ligament. J Am Vet Med Assoc.

[37] Kalff S, Meachem S, Preston C (2011). Incidence of medial meniscal tears after arthroscopic assisted tibial plateau leveling osteotomy. Vet Surg.

